# The G2A receptor (GPR132) contributes to oxaliplatin-induced mechanical pain hypersensitivity

**DOI:** 10.1038/s41598-017-00591-0

**Published:** 2017-03-27

**Authors:** Stephan W. Hohmann, Carlo Angioni, Sorin Tunaru, Seungkyu Lee, Clifford J. Woolf, Stefan Offermanns, Gerd Geisslinger, Klaus Scholich, Marco Sisignano

**Affiliations:** 1 Institute of Clinical Pharmacology, pharmazentrum frankfurt/ZAFES, University Hospital, Goethe-University, D-60590 Frankfurt am Main, Germany; 2 Department of Pharmacology, Max-Planck-Institute for Heart and Lung Research, 61231 Bad Nauheim, Germany; 3 F. M. Kirby Neurobiology Center, Children’s Hospital Boston, and Department of Neurobiology, Harvard Medical School, Boston, MA 02115 USA; 4 Fraunhofer Institute for Molecular Biology and Applied Ecology – Project Group Translational Medicine and Pharmacology (IME-TMP), Frankfurt am Main, Germany

## Abstract

Chemotherapy-induced peripheral neuropathic pain (CIPN) is a common and severe debilitating side effect of many widely used cytostatics. However, there is no approved pharmacological treatment for CIPN available. Among other substances, oxaliplatin causes CIPN in up to 80% of treated patients. Here, we report the involvement of the G-protein coupled receptor G2A (GPR132) in oxaliplatin-induced neuropathic pain in mice. We found that mice deficient in the G2A-receptor show decreased mechanical hypersensitivity after oxaliplatin treatment. Lipid ligands of G2A were found in increased concentrations in the sciatic nerve and dorsal root ganglia of oxaliplatin treated mice. Calcium imaging and patch-clamp experiments show that G2A activation sensitizes the ligand-gated ion channel TRPV1 in sensory neurons via activation of PKC. Based on these findings, we conclude that targeting G2A may be a promising approach to reduce oxaliplatin-induced TRPV1-sensitization and the hyperexcitability of sensory neurons and thereby to reduce pain in patients treated with this chemotherapeutic agent.

## Introduction

Chemotherapy-induced peripheral neuropathic pain (CIPN) is a severe adverse event of cytostatic drugs during cancer therapy. CIPN is one of the major reasons for delay or discontinuation of chemotherapy and is therefore responsible for decreased chemotherapeutic efficacy and loss of quality of life^1, 2^. Currently, there are no drugs available for the prevention or effective treatment of CIPN^1, 3^. One of the most frequently used agents causing CIPN is the platinum derivate oxaliplatin. It is widely used as first-line treatment for e.g. colorectal carcinomas and induces CIPN in up to 80% of patients^4^. Oxaliplatin can cause two different types of pain, an acute pain syndrome in the beginning of treatment which usually subsides within a few days, and with later onset, a chronic distal sensory neuropathy. These symptoms manifest in the extremities of the body and may proceed along the limbs over the course of repetitive treatment^4, 5^.

The molecular mechanisms underlying oxaliplatin-induced peripheral neuropathic pain are still unclear, but it is known that ligand-gated ion channels such as transient receptor potential (TRP) channels may contribute to the pathology of CIPN. Under physiological conditions TRP channels serve for the perception of external stimuli like heat or capsaicin from chilli pepper (TRPV1), low pH or allyl isothiocyanate from wasabi (TRPA1), cool temperatures or menthol from peppermint (TRPM8) as well as osmotic and mechanical stimulations (TRPA1, TRPV4, TRPC3 and TRPC6)^6^. Under pathophysiological conditions TRP channels are sensitized, which causes a reduction of their activation threshold and thereby increased sensitivity to painful (hyperalgesia) or normally non-painful (allodynia) stimuli^7^. These sensitization effects on TRP channels can be mediated by the activation of G-protein coupled receptors (GPCRs). This activation of G-protein-dependent signaling pathways leads to protein kinase A (PKA)- and PKCε-mediated phosphorylation of TRP channels causing a reduction of their activation threshold^8, 9^. Increased sensitization of TRPV1, TRPA1 and TRPV4 have been demonstrated to contribute to mechanical hypersensitivity and cold allodynia in chemotherapy-induced peripheral neuropathic pain^10–12^.

Treatment with cytostatics, such as oxaliplatin, induces sensitization mechanisms in the peripheral nervous system, leading to increased activity of peripheral sensory neurons and increased pain perception^1^. Interestingly, the G-protein coupled receptor G2A (GRP132) is described to be expressed in TRPV1-expressing peripheral sensory neurons^13^ which play an important role in the pathophysiology of pain and in the development of CIPN^1^. The expression of G2A has been demonstrated to be upregulated by different DNA-damaging agents^14^. We therefore hypothesize that treatment with oxaliplatin induces G2A-expression in peripheral sensory neurons and thereby contributes to sensitization of nociceptive neurons and subsequently to oxaliplatin-induced neuropathic pain.

## Results

### G2A-deficient mice have reduced oxaliplatin-induced mechanical hypersensitivity

**Figure 1:**
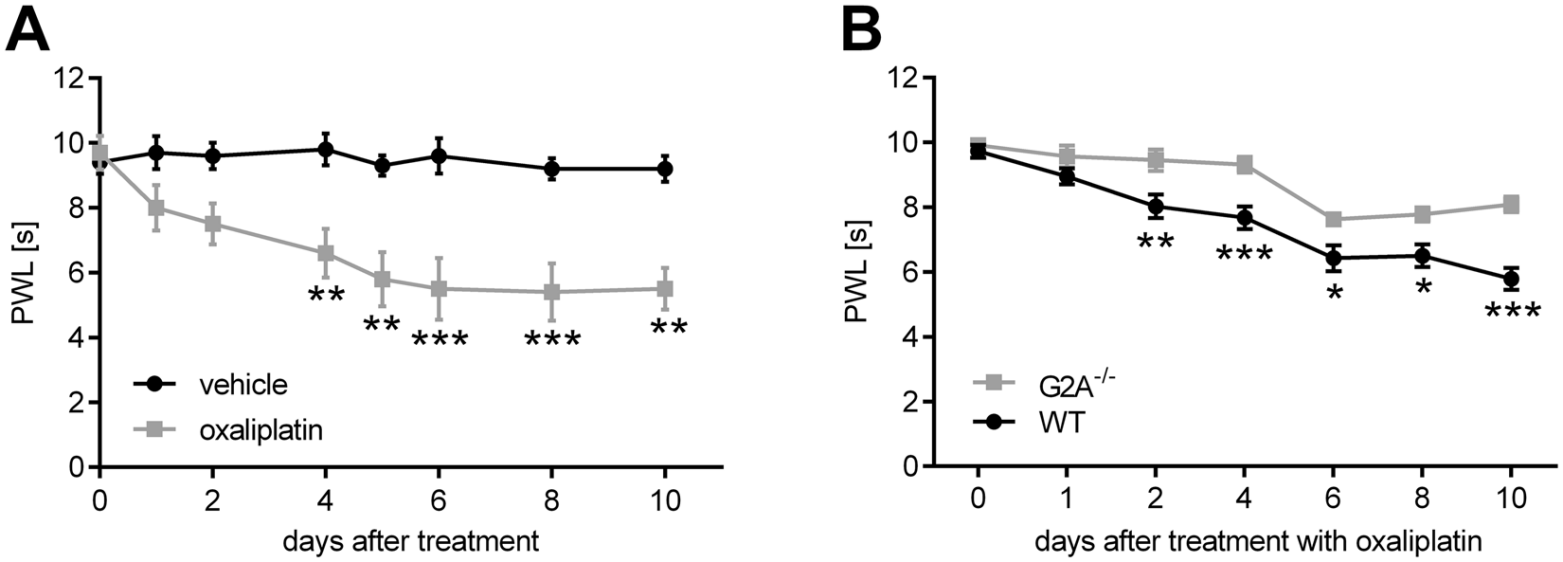
G2A-deficient mice have reduced oxaliplatin-induced mechanical pain thresholds. (**A**) Mechanical pain thresholds of mice after i.p.-injection of oxaliplatin (3 mg/kg) compared to vehicle (0.9% v/v DMSO) using a dynamic plantar aesthesiometer over ten days. Shown is the mean ± SEM of paw withdrawal latency (PWL) of n = 8 mice per group. (**B**) Mechanical pain thresholds of wildtype (WT) compared to G2A-deficient mice after treatment with oxaliplatin (3 mg/kg) using a dynamic plantar aesthesiometer over ten days. Shown is the mean ± SEM of PWL of n = 8 mice per group; *p < 0.05 **p < 0.01 ***p < 0.001 two-way ANOVA with Bonferroni post-hoc test.

First we tested the effect of oxaliplatin (3 mg/kg oxaliplatin^10^ i.p.) on the mechanical pain thresholds of wild type C57BL/6N mice over ten days using a dynamic plantar aesthesiometer. Oxaliplatin-treated mice showed significantly reduced paw withdrawal latencies starting at day four with maximal effects between days eight to ten (Fig. 1A). Since it is known that oxaliplatin does not induce heat hyperalgesia in animals or in patients^15, 16^, we did not determine thermal thresholds in mice following oxaliplatin treatment. Instead, we next investigated the effect of oxaliplatin treatment on the mechanical pain thresholds of G2A-deficient mice. To verify the absence of G2A, we performed qRT-PCR on DRGs of G2A-deficient mice and could not measure any detectable transcript (data not shown). In behaviour experiments we observed significantly reduced mechanical hypersensitivity over ten days after treatment as compared to wild type mice (Fig. 1B).

**Figure 2:**
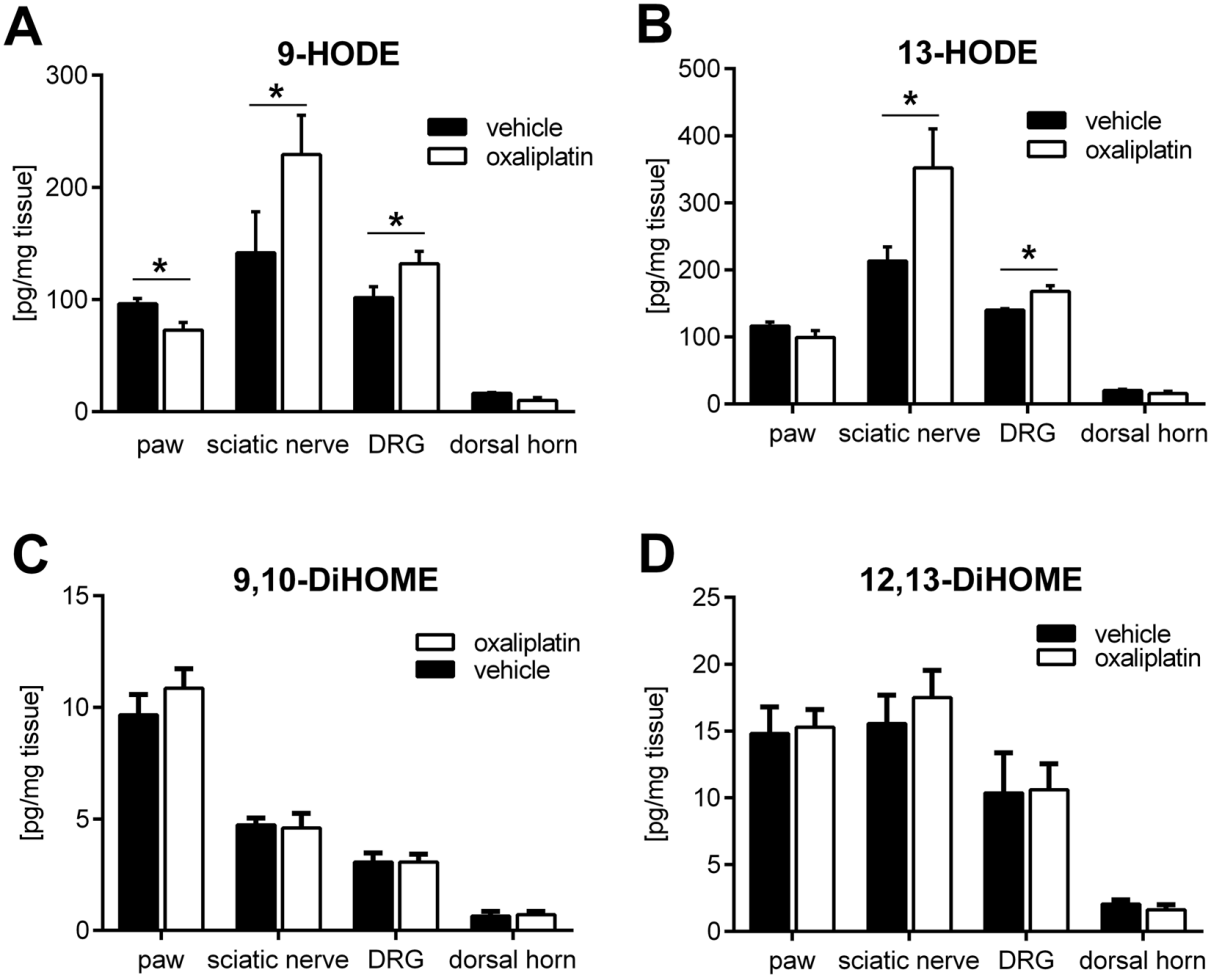
Increased oxidized lipid concentrations in sciatic nerve and DRGs after ten days’ treatment with oxaliplatin. Concentrations of 9-HODE (**A**), 13-HODE (**B**), 9,10-DiHOME (**C**) and 12,13-DiHOME (**D**) using LC-MS/MS measurements from plantar paw, sciatic nerve, DRGs and dorsal horn tissue of mice treated with vehicle (black bars; 0.9% v/v DMSO) or oxaliplatin (white bars; 3 mg/kg) ten days after injection. Data represent mean ± SEM from n = 5 mice per group; *p < 0.05 unpaired student’s t-test.

It has been shown previously that oxidized lipids, predominately oxidized linoleic acid metabolites, are agonists of G2A^17^. To investigate whether or not endogenous G2A-agonists such as 9-HODE and 13-HODE are synthesized in response to oxaliplatin treatment, we determined the levels of oxidized lipids via LC-MS/MS ten days after treatment with oxaliplatin (3 mg/kg) or vehicle (0.9% DMSO) in plantar paw tissue, sciatic nerve, L1-L6 DRGs and the corresponding section of the dorsal horn of mice. We quantified the concentrations of oxidized linoleic acid metabolites (OLAMs) hydroxyoctadecadienoic acids (HODEs), dihydroxyoctadecenoic acids (DiHOMEs), and epoxyoctadecenoic acids (EpOMEs) that have been reported to be synthesized during inflammatory pain previously^18^ and found that the concentrations of 9-HODE (Fig. 2A) and 13-HODE (Fig. 2B) were significantly increased in the sciatic nerve and DRGs, and concentrations of 9-HODE decreased in plantar paw tissue, after treatment with oxaliplatin. There were no changes in concentrations observed in the dorsal horn of the spinal cord. The concentrations of 9,10-DiHOME (Fig. 2C) and 12,13-DiHOME (Fig. 2D) were not altered in any of the tissues analysed and the concentrations of 9,10-EpOME and 12,13-EpOME were below the detection limit.

### 9-HODE-induced sensitization of TRPV1 in sensory neurons is mediated by G2A-induced PKC-activation

**Figure 3:**
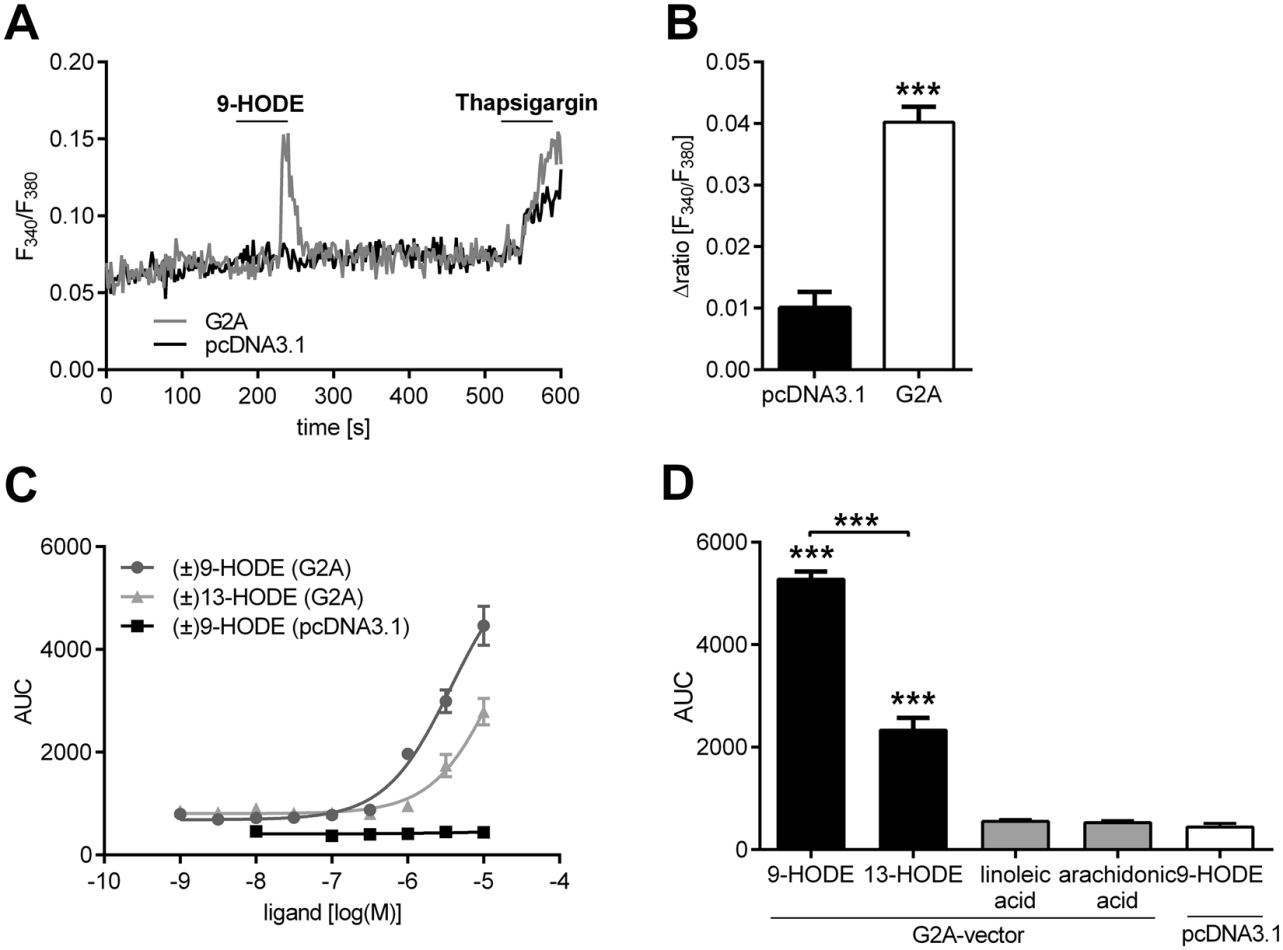
9-HODE activates the G2A-receptor (GRP132). (**A**,**B**) Shown are calcium concentrations of HEK-293 cells transfected with G2A-vector or pcDNA3.1 (empty vector) stimulated with 9-HODE (1 µM). Thapsigargin (1 µM) was used as positive control. (**A**) Shows a representative trace and (**B**) the quantitative analysis presented as mean ± SEM with n = 32–46 cells per condition. ***p < 0.001 unpaired student’s t-test. (**C**) Dose-response analysis of COS-1 cells co-transfected with aequorin- and G2A-expressing vector or pcDNA3.1 (empty vector). Seen is the calcium flux as area under curve (AUC) at different 9-HODE and 13-HODE concentrations [10^−9^–10^−5^ M]. Data are presented as mean ± SEM from n = 3 measurements. (**D**) COS-1 cells cotransfected with aequorin- and G2A-expressing vector stimulated with 9-HODE (10 µM), 13-HODE (10 µM), linoleic acid (30 µM) and arachidonic acid (30 µM). Shown is the calcium flux as mean of area under curve (AUC) ± SEM with n = 3 measurements; ***p < 0.001 one-way ANOVA with Bonferroni post-hoc test.

9-HODE is one of the most potent agonists for the G-protein coupled receptor G2A^19^. To investigate whether or not 9-HODE may act on the G2A receptor, we used HEK-293 cells overexpressing G2A and observed increased intracellular calcium concentrations after stimulation with 9-HODE (Fig. 3A and B). To verify this, we used another heterologous expression (COS-1 cells with a sensitive aequorin-calcium-system) and observed that the activation of G2A by 9-HODE is concentration-dependent (Fig. 3C). To investigate whether the activation of G2A is specific for 9-HODE, we stimulated G2A-overexpressing cells with 13-HODE, as well as the precursor linoleic acid and arachidonic acid. 13-HODE activates the G2A to a lesser extent as compared to 9-HODE, but we did not observe any increase in intracellular calcium concentration after stimulation with linoleic acid or arachidonic acid (Fig. 3D).

**Figure 4:**
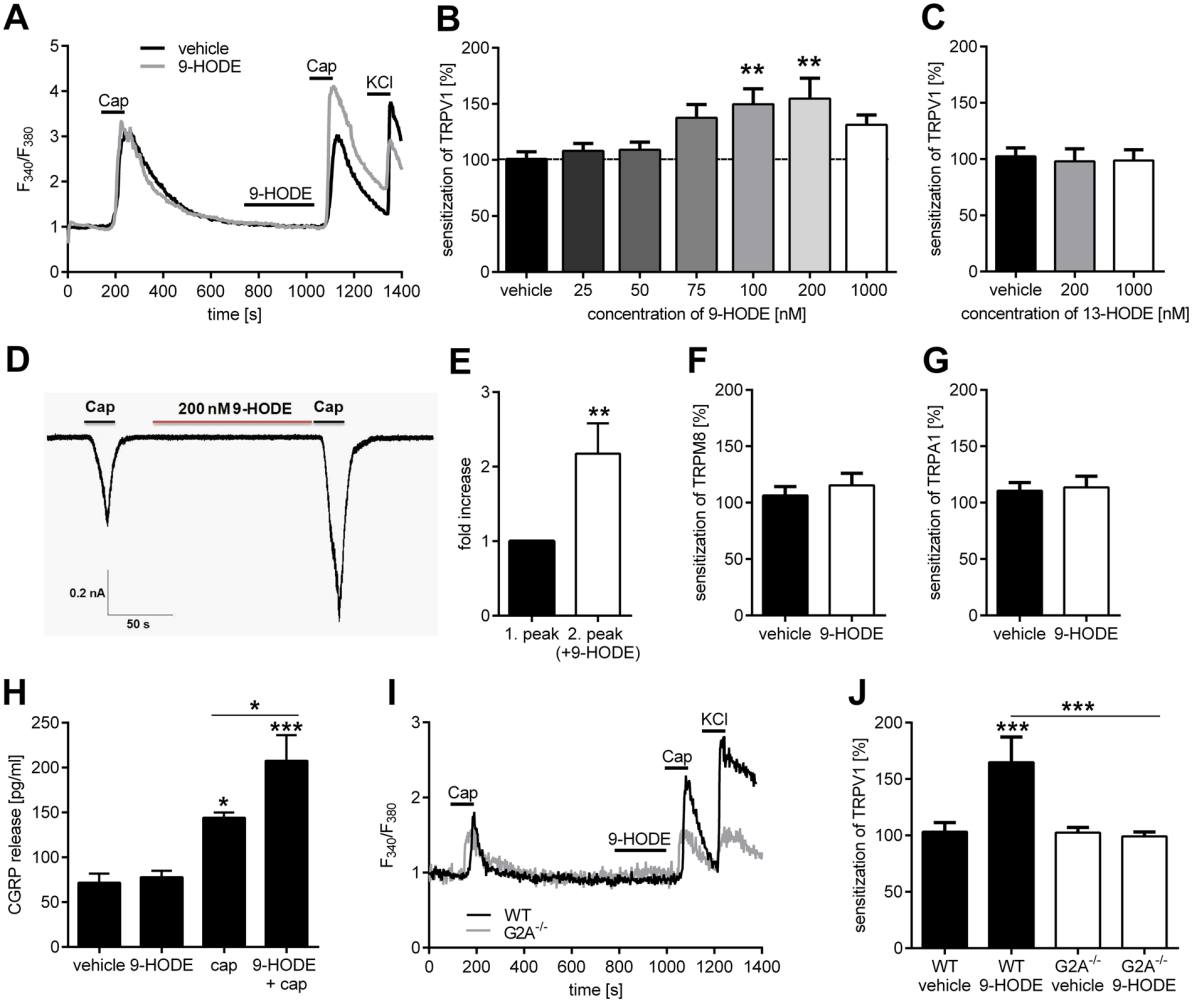
9-HODE-dependent activation of G2A causes TRPV1 sensitization. (**A**) Stimulation of TRPV1 of adult DRG neurons two times with capsaicin (Cap; 200 nM, 30 s). Incubation with 9-HODE (200 nM, 120 s) before the second stimulus leads to an increased calcium flux compared to the first stimulus (Δ_ratio_). Neurons were identified by responses to KCl (50 mM, 10 s). Shown is a representative trace. (**B**) 9-HODE dose-dependently sensitizes the TRPV1 channel of DRG neurons. Shown is the Δ_ratio_ of the first and second capsaicin stimulus at 9-HODE concentrations ranging from 0 to 1000 nM. Data are presented as mean ± SEM with n = 44–99 neurons per concentration; **p < 0.01 one-way ANOVA with Bonferroni post-hoc test. (**C**) 13-HODE (200 nM, 120 s) does not increase the Δ_ratio_ of TRPV1-dependent calcium flux in DRG neurons (n = 30–83). (**D**) Sample trace of whole-cell voltage clamp measurement of DRG neurons treated with capsaicin (1 µM, 15 s) twice. Before the second cap-stimulus, cells were incubated with 9-HODE (200 nM, 3 min). (**E**) Normalized response to first and second capsaicin-stimulus with cells treated like in (**D**) shown as mean ± SEM with n = 11 neurons; **p < 0.01 unpaired student’s t-test. Effect of 9-HODE on TRPM8 (**F**) of DRG neurons stimulated with menthol (100 µM, 30 s) and TRPA1 (**G**) stimulated with allyl isothiocyanate (50 µM, 30 s). The Δ_ratio_ is depicted compared to vehicle (DMSO) with n = 20–33 neurons per condition. (**H**) CGRP release from DRG neurons stimulated with 9-HODE (1 µM, 10 min), capsaicin (400 nM, 10 min) or both together. Data shown as mean ± SEM from n = 6–8 mice per condition; *p < 0.05 ***p < 0.001 one-way ANOVA with Bonferroni post-hoc test. (**I**,**J**) Incubation of adult DRG neurons with 9-HODE (200 nM, 120 s) prior to the second capsaicin stimulus (Cap; 200 nM, 30 s) leads to an increased calcium flux compared to the first stimulus (Δ_ratio_) in DRG neurons of wildtype (WT) but not in G2A-deficient mice. Neurons were identified by responses to KCl (40 mM, 10 s). (**I**) Representative trace. (**J**) The quantitative analysis presented as mean ± SEM with n = 46–110 cells per condition; ***p < 0.001 one-way ANOVA with Bonferroni post-hoc test.

The TRPV1 channel is one of the prominent members of ligand-gated ion channels in the perception of pain^7^. It was recently shown, that TRPV1 takes a crucial role during the cause of CIPN through receptor-signaling^20^. This type of signaling is commonly mediated via activation of GPCRs^21, 22^. We therefore investigated whether or not 9-HODE or 13-HODE can sensitize TRPV1. DRG neurons from mice were stimulated with capsaicin and incubated with vehicle, 9-HODE or 13-HODE for two minutes before a second capsaicin stimulus was applied (Fig. 4A). The Δ_ratio_ of the first and second peak was calculated to determine whether or not a TRPV1 sensitization occurred (Fig. 4B). We found that 9-HODE concentration-dependently induced sensitization of TRPV1 at concentrations between 100 nM and 200 nM (Fig. 4B). In contrast, 13-HODE had no sensitizing effect on capsaicin-induced TRPV1 activation (Fig. 4C) probably because the required concentrations to activate G2A are considerably higher than the required 9-HODE concentrations. Whole-cell voltage clamp measurement of DRG neurons confirmed that 9-HODE induces TRPV1 sensitization; the capsaicin-induced inward current of cells pre-treated with 9-HODE was significantly increased compared with vehicle pre-treated cells (Fig. 4D and E). 9-HODE also increased capsaicin-dependent CGRP-release significantly in DRG neurons (Fig. 4H). To analyse whether other TRP channels can be affected by 9-HODE, we investigated TRPM8 and TRPA1 sensitization caused by 9-HODE. However, 9-HODE showed no sensitizing effects on TRPM8 (activated with 100 µM menthol; Fig. 4F) or TRPA1 (activated with 50 µM AITC; Fig. 4G). To determine whether or not the effects of 9-HODE towards TRPV1 sensitization are G2A-dependent, we compared primary DRG cultures of wild type and G2A-deficient mice and found that the 9-HODE-induced TRPV1 sensitization was absent in DRG cultures from G2A-deficient mice (Fig. 4I,J). These data suggest that 9-HODE activates the G2A receptor which may subsequently lead to TRPV1 sensitization in mice.

**Figure 5:**
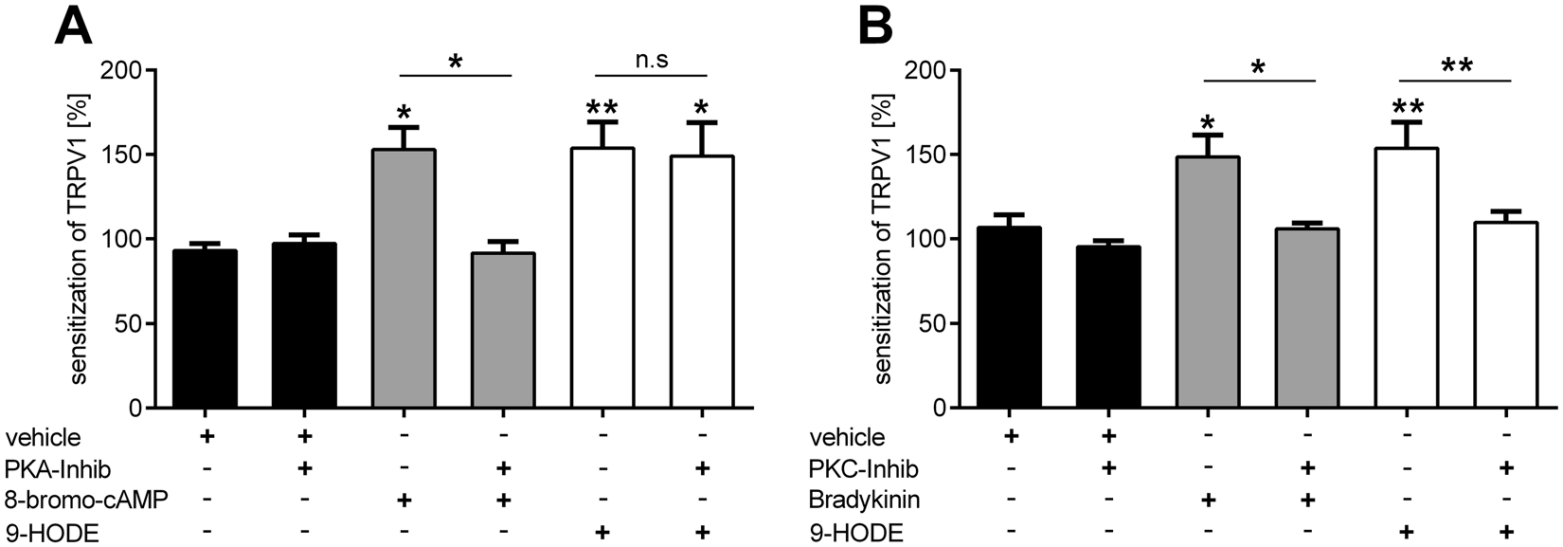
TRPV1 sensitization via 9-HODE-dependent activation of G2A is mediated by PKC but not PKA. (**A**) Sensitization of TRPV1 via 9-HODE (200 nM, 120 s) of adult DRG neurons is not influenced by the PKA-Inhibitor H89 (10 µM, 45 min before measurement) in calcium imaging experiments. As control for PKA-dependent TRPV1 sensitization 8-bromo-cAMP (40 µM, 120 s) was used. Data shown as mean ± SEM with n = 20–69 cells per condition. (**B**) The PKC-Inhibitor GF109203X (1 µM, 45 min before measurement) suppresses the 9-HODE-dependent sensitization of TRPV1. Bradykinin (500 nM, 120 s) was used as positive control for PKC-dependent sensitization of TRPV1. Data is shown as mean ± SEM with n = 45–90 cells per condition; *p < 0.05 **p < 0.01 one-way ANOVA with Bonferroni post-hoc test.

TRPV1 sensitization can be mediated by PKA or PKC^23, 24^. To investigate the signaling pathway between G2A and TRPV1 in DRG neurons, we determined which protein kinases are involved by using inhibitors for PKA (H89; Fig. 5A) and PKC (GF-109203X; Fig. 5B) and tested their effects on the 9-HODE-induced sensitization of TRPV1. Incubation of DRG neurons with the PKA-inhibitor H89 inhibited the PKA-dependent TRPV1 sensitization caused by 8-bromo-cAMP but failed to supress the 9-HODE-induced TRPV1 sensitization (Fig. 5A). However, the PKC-inhibitor GF109203X prevented TRPV1 sensitization caused by 9-HODE (Fig. 5B). Bradykinin was used as positive control for PKC-dependent TRPV1 sensitization. Thus, we conclude that TRPV1 sensitization caused by G2A is PKC-dependent.

### TRPV1- and G2A-deficient mice have reduced pain in response to 9-HODE and oxaliplatin treatment

**Figure 6:**
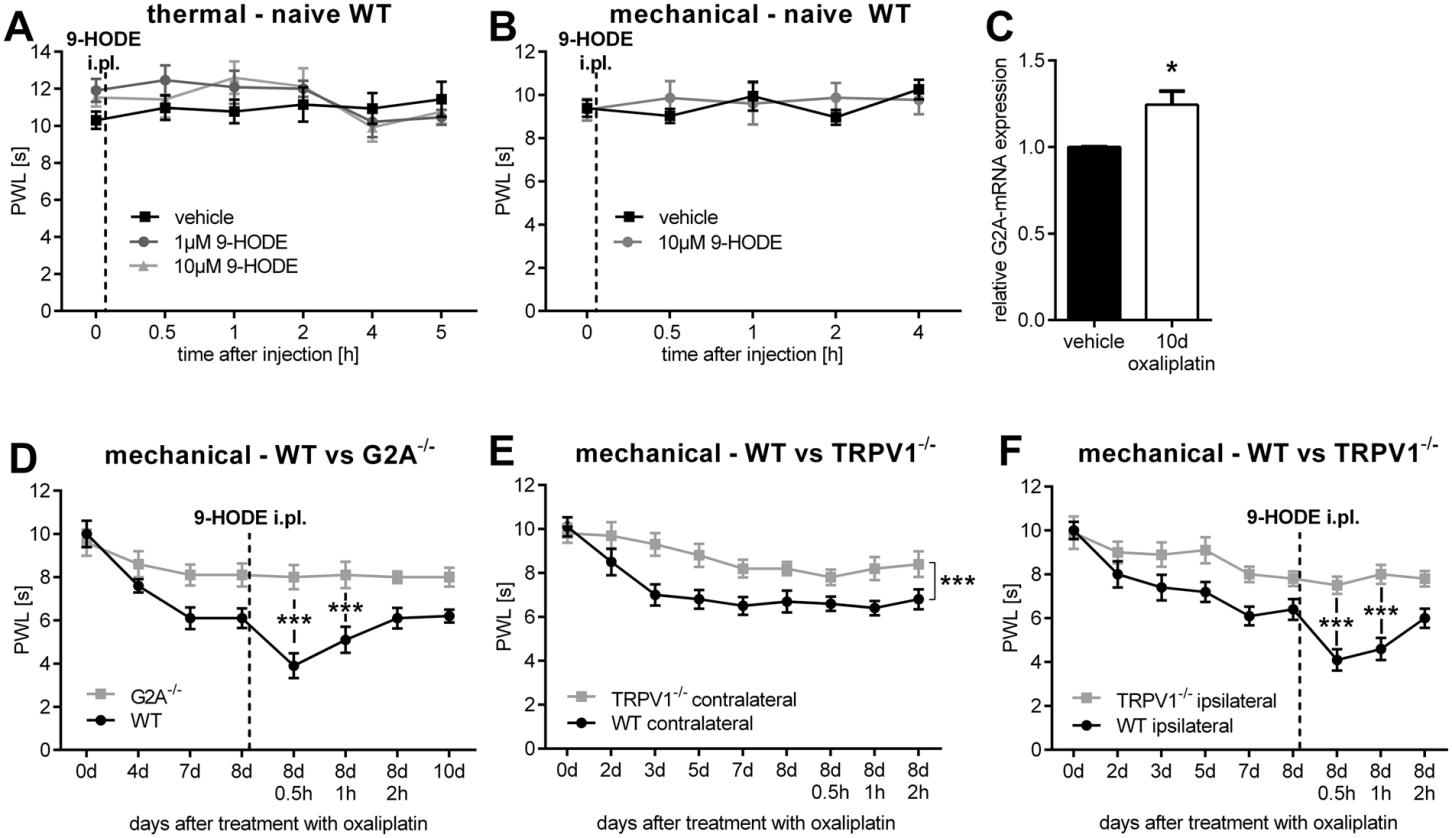
G2A-deficient and TRPV1-deficient mice have reduced 9-HODE-dependent mechanical pain during oxaliplatin treatment. (**A**) Thermal pain thresholds of naive wildtype mice after i.pl. (intraplantar) injection of 9-HODE (1 µM and 10 µM) into the left hind paw using a Radiant Heat Test. Shown is the mean ± SEM of PWL of n = 8 mice per group. (**B**) Mechanical pain thresholds of naive wildtype mice after i.pl. (intraplantar) injection of 9-HODE (10 µM) into the left hind paw using a dynamic plantar aesthesiometer. Shown is the mean ± SEM of PWL of n = 6 mice per group. (**C**) G2A-mRNA expression in DRG of mice treated with oxaliplatin (3 mg/kg) for ten days compared to vehicle treated mice. Data presented as mean ± SEM with n = 4 mice per group; *p < 0.05 unpaired student’s t-test. (**D**) Mechanical pain thresholds of wildtype compared to G2A-deficient mice after treatment with oxaliplatin (3 mg/kg) over eight days. Then 9-HODE (10 µM) was injected i.pl. into the left hind paw and PWL was measured again after 0.5 h, 1 h and 2 h. Shown is the mean ± SEM of PWL of the 9-HODE treated paw of n = 7 mice per group; ***p < 0.001 two-way ANOVA with Bonferroni post-hoc test. (**E**) Mechanical pain thresholds of wildtype mice (WT) compared to TRPV1-deficient mice after treatment with oxaliplatin (3 mg/kg) using a dynamic plantar aesthesiometer over eight days. Shown is the mean ± SEM of PWL of the contralateral paw (untreated) of n = 7 mice per group; ***p < 0.001 two-way ANOVA. (**F**) Mechanical pain thresholds of wildtype mice compared to TRPV1-deficient mice after treatment with oxaliplatin (3 mg/kg). At day eight, 9-HODE (10 µM) was injected i.pl. into the ipsilateral paw and PWL was measured again after 0.5 h, 1 h and 2 h. Shown is the mean ± SEM of PWL of the ipsilateral paw (9-HODE treated) of n = 7 mice per group; ***p < 0.001 two-way ANOVA with Bonferroni post-hoc test.

To test the hypothesis that G2A mediates the pronociceptive effects of 9-HODE *in vivo*, we first injected 9-HODE intraplantar into the left hind paw of naive wildtype mice and assessed their nociceptive behaviour. We found no change in the thermal (Fig. 6A) and mechanical (Fig. 6B) paw withdrawal latencies of these mice. Since G2A is known to be upregulated in response to cellular stress^17, 25^, we compared its expression in naive mice and oxaliplatin-treated mice. We found that the expression of G2A-mRNA in DRGs was significantly increased ten days after oxaliplatin injection (Fig. 6C). Therefore, we tested in the next step the effect of 9-HODE on mechanical paw withdrawal latencies eight days after oxaliplatin injection in wild type and G2A-deficient mice, since we found reduced paw withdrawal latencies with maximal effects between days eight to ten. Indeed, we observed a strong reduction of mechanical pain thresholds in wildtype mice but not in G2A-deficient mice (Fig. 6D). Finally, we studied the effect of TRPV1-deletion on oxaliplatin-induced paw withdrawal latencies. To verify the absence of TRPV1, we performed qRT-PCR on DRGs of TRPV1-deficient mice and could not detect any G2A transcript (data not shown). Although TRPV1 is not a direct mechanical transducer channel, it is responsible for mechanical allodynia due to central sensitization^26^. In behaviour experiments, TRPV1-deficient mice displayed reduced mechanical hypersensitivity over the complete time period of eight days after treatment with oxaliplatin compared with wild type mice (Fig. 6E). Furthermore, intraplantar injection of 9-HODE into the left hind paw (ipsilateral) eight days after oxaliplatin-injection induced a strong reduction of mechanical paw withdrawal latencies in wild type mice but not in TRPV1-deficient mice (Fig. 6F). We conclude that G2A is the receptor for 9-HODE and mediates the effects of this oxidized lipid on TRPV1 in primary sensory neurons during the course of oxaliplatin-induced pain in mice.

## Discussion

Like vinca alkaloids (e.g. vincristine), taxanes (paclitaxel) or proteasome inhibitors (bortezomib), the platinum-derivative oxaliplatin is an antineoplastic drug which induces severe pain and neuropathy^15^. It is widely used as first-line treatment for colorectal carcinomas and causes CIPN in up to 80% of patients^4^. Although CIPN involves inflammatory processes, the key enzymes and signaling molecules seem to differ as compared to classical inflammatory pain models. Inside the cells, oxaliplatin is degraded to oxalate and platinum. The oxalate may cause disturbance of neuronal membrane potentials and of the activity of voltage-gated ion channels by chelating intracellular calcium^27, 28^. Furthermore, oxaliplatin induces cell damage, leading to the activation of p38-mitogen activated protein kinase (MAPK) pathways possibly induced by DNA damage^29, 30^. The production of reactive oxygen species (ROS) in neuronal cells has also been observed in response to oxaliplatin-treatment and seems to be induced by mitochondrial dysfunction^31, 32^. The combination of these mechanisms causes cellular stress responses in peripheral sensory neurons which contribute to oxaliplatin-induced CIPN. Indeed, the G2A-receptor has been described as stress-inducible GPCR that functions at the G2/M checkpoint of the cell cycle and its expression is induced by several classes of DNA-damaging agents^14^. We found the G2A-mRNA expression increased after treatment of wild type mice with oxaliplatin, thus pointing towards an oxaliplatin-induced induction of G2A gene expression in sensory neurons.

The G2A-receptor is classified as a member of the proton sensing GPCR subfamily, but its ability to act as a pH-sensor is less distinct than the other members of this family (OGR1, GPR4, TDAG8)^33^. Some histidine residues in the N-terminal extracellular regions and extracellular loops were found to be critical for proton-sensing activity and are exchanged by other basic amino acids like lysine and arginine in the G2A-receptor^34^. However, the G2A acts as a receptor for endogenous polyunsaturated fatty acids like OLAMs^19^. We show that the concentrations of the endogenous G2A agonists 9-HODE and 13-HODE are increased in the DRGs and sciatic nerve, but not in the spinal cord of oxaliplatin-treated mice. We also observed decreased concentrations of 9-HODE in plantar paw tissue. The increased production of 9-HODE in sciatic nerve and DRG tissue and the decreased concentrations of this lipid in plantar paw tissue, which is a mixture of various cell types, indicate, that synthesis of 9-HODE is restricted to neuronal tissue during oxaliplatin-induced CIPN. This supports previous reports of neuronal 9-HODE synthesis in NGF-induced pain^18^.

The oxidized lipid 9-HODE activates G2A^19^ and here we confirm this in a dose-response analysis. We also observed that 13-HODE activates G2A, although the concentrations required for this activation were around six-fold higher than the required concentrations of 9-HODE and we do not think that these are physiologically occurring concentrations. Therefore, we focused on 9-HODE-dependent G2A-activation in this study, as 9-HODE seems to be a more potent G2A agonist. Injection of 9-HODE in untreated naive mice did not evoke thermal or mechanical pain, while mice treated with oxaliplatin showed enhanced mechanical hypersensitivity after injection of 9-HODE. These findings suggest the necessity of an induction of the G2A-receptor in neurons by pathological stimuli before it can carry out its pro-nociceptive activity.

Stressful situations like chemotherapy treatment and subsequent damage of sensory neurons as well as peripheral nerves may induce both ligand production (9-HODE) and receptor expression (G2A). Previous studies described that 9-HODE is a direct activator of TRPV1 at high concentrations (100 µM)^35^, although direct activation of TRPV1 by 9-HODE is controversial^36, 37^. We found that 9-HODE sensitizes TRPV1 at concentrations of 200 nM which is several magnitudes lower than the direct effects and is also considerably lower as compared to other TRPV1 sensitizing lipids (e.g. PGE_2_ 1 µM^8^, 20-HETE 10–30 µM^38^). We also observed that 9-HODE increases TRPV1-dependent release of the neuropeptide CGRP. Together with our observation of decreased mechanical hypersensitivity in TRPV1-deficient mice during oxaliplatin treatment as well as after injection of 9-HODE, these findings indicate, that the role of 9-HODE-dependent G2A activation during oxaliplatin treatment is predominantly driven by TRPV1-dependent effects in sensory neurons. Moreover, previous studies described the contribution of TRPA1 and TRPM8 to oxaliplatin induced neuropathic pain^10, 39^, however, we did not observe any 9-HODE-dependent sensitization effect on TRPA1 or TRPM8.

Although TRPV1 is not a direct mechanical transducer channel, it may be responsible for oxaliplatin-induced mechanical hypersensitivity due to central sensitization. It was previously shown that increased TRPV1-activity caused by capsaicin and increased nociceptive input in the spinal cord leads to central sensitization which can subsequently cause mechanical hypersensitivity in the periphery by sensitizing mechanically sensitive Aδ-fibers^26^. Indeed, the involvement of TRPV1 in chemotherapy-induced pain has been indicated before^12^. Moreover, in a recently published study^20^, pharmacological inhibition of TRPV1 with a selective antagonist strongly reduced paclitaxel-induced mechanical hypersensitivity, thus indicating the importance of TRPV1 in chemotherapy-induced mechanical pain hypersensitivity.

According to our results, the G2A-receptor in DRGs causes PKC-activation and subsequent TRPV1 sensitization in peripheral sensory neurons. This is in line with previous reports concerning the coupling of G2A^40^. Furthermore, the G2A receptor is described to be expressed in 32% of total DRG neuron population and co-localized with TRPV1 in 46% of all G2A-positive neurons^13^, thus supporting our hypothesis that G2A sensitizes TRPV1 in a subset of sensory neurons. We therefore suggest, that targeting the G2A receptor and blocking the 9-HODE-G2A-PKC-TRPV1 pathway might offer a novel approach for the treatment of oxaliplatin-induced neuropathic pain.

## Methods

### Ethics Statement

All experiments with animals involved were approved by the local Ethics Committees for Animal Research (Darmstadt) with the permit numbers F95/47 and FK/1046 and were performed according to the recommendations of the Guide of the Care and Use of Laboratory Animals of the National Institutes of Health^41^. All efforts were made to minimize suffering.

### Animals

In all behavioral tests the experimenter was unaware of the treatment or the genotype of the mice. For all behavioral experiments, we used 6–12 weeks old C57BL/6N mice purchased from commercial breeding companies (Charles River, Sulzfeld, Germany, Janvier, Le Geneset-Saint-Isle, FR). G2A-deficient mice were generated by the lab of Owen Witte, University of California at San Francisco^42^ and TRPV1-deficient mice were generated by the lab of David Julius, University of California, Los Angeles^43^. Both lines were obtained from Jackson laboratories. All preclinical pain models were in accordance with the suggestions from the Preclinical Pain Research Consortium for Investigating Safety and Efficacy (PPRECISE) Working Group^44^.

### Treatments

All mice were randomized to the treatment. For the intraplantar (i.pl.) treatment with 10 µM 9-HODE ((±)-9-hydroxy-10E,12Z-octadecadienoic acid) (Cayman), a volume of 10 µl was injected in the mid-plantar area of the hindpaw. For the intraperitoneal (i.p.) treatment, 100 µl of Oxaliplatin (3 mg/kg, Tocris) were injected into the peritoneum. The control group animals were treated with the corresponding volumes of 0.9% DMSO (v/v) solution.

### Behavioral Testing

#### Mechanical pain

To determine the mechanical thresholds of mice, a Dynamic Plantar Aesthesiometer (Ugo Basile, Comerio, Italy) was used. Mice were put on an elevated grid in test cages at least 2 h before the measurement to allow accommodation. Then, baseline measurement was performed before the treatment with Oxaliplatin or 9-HODE. Therefore, a steel rod pushed against the plantar side of the hind paw with linear ascending force (0–5 g over 10 s, in 0.5 g/s intervals) until a fast withdraw of the paw was observed. The time between the first contact of the steel rod and the paw withdraw was measured in seconds (paw withdraw latency) and a cut-off time of 20 s was set^45^.

#### Thermal pain

To determine the thermal thresholds, mice were kept in test cages on a warmed glass plate (32 °C) for at least 2 h on the first day to allow accommodation. Then the mid-plantar region of the paws was stimulated with a radiant heat device, consisting of a high-intensity projector lamp, until withdrawal occurred, as described before^46^. The non-injected and injected paws were measured alternately in intervals of 5–10 min.

### Determination of Lipids from Tissue Samples by LC-MS/MS

The spinal cord from L4-L6, both sciatic nerves, dorsal root ganglia (DRGs) from L1-L6 as well as the plantar paw tissue were prepared from adult mice.

#### Lipid extraction and standards

Stock solutions with 2500 ng/ml of the analytes 9-HODE ((±)-9-hydroxy-10E,12Z-octadecadienoic acid), 13-HODE ((±)-13-hydroxy-9Z,11E-octadecadienoic acid), 9,10-EpOME ((±)9,10-epoxy-12Z-octadecenoic acid), 12,13-EpOME ((±)12(13)epoxy-9Z-octadecenoic acid), 9,10-DiHOME ((±)9,10-dihydroxy-12Z-octadecenoic acid) and 12,13-DiHOME ((±)12,13-dihydroxy-9Z-octadecenoic acid), as well as their internal standards (9- and 13-HODE-d4, 9,10- and 12,13-EpOME-d4, 9,10- and 12,13-DiHOME-d4) were prepared in methanol. Working standards were obtained by further dilution with a concentration range of 0.25–2500 ng/ml for the analyte.

Lipids were extracted using liquid–liquid extraction. Therefore, homogenated tissue was extracted twice with 600 µl of ethyl acetate. The combined organic phases were removed at a temperature of 45 °C under a gentle stream of nitrogen. The residues were reconstituted with 50 µl of methanol/water/butylated hydroxytoluene (BHT) (50:50:10^−3^, v/v/v) and then centrifuged for 2 min at 10,000 g, and transferred to glass vials waiting for analysis.

#### Instrumentation for lipid measurement

The LC-MS/MS system consisted of a QTrap 5500 (Sciex, Darmstadt, Germany) equipped with a Turbo-V source operating in negative electrospray ionization mode, an Agilent 1200 binary HPLC pump and degasser (Agilent, Waldbronn, Germany), and an HTC Pal autosampler (CTC analytics, Zwingen, Switzerland). High-purity nitrogen for the mass spectrometer was produced by a NGM 22-LC-MS nitrogen generator (cmc Instruments, Eschborn, Germany).

For the chromatographic separation of oxidized lipids, a Gemini NX C18 column and precolumn were used (150 × 2 mm inner diameter, 5 µm particle size, and 110 Å pore size; Phenomenex, Aschaffenburg, Germany). A linear gradient was used at a flow rate of 0.5 ml/min with a total run time of 17.5 min. Mobile phase A consist of water:ammonia (100:0.05, v/v), and mobile phase B of acetonitrile:ammonia (100:0.05, v/v). The gradient changed from 85% A to 10% within 12 min. These conditions were held for 1 min. Then, the mobile phase shifted back to 85% A within 0.5 min and it was maintained for 4 min to re-equilibrate the column.

20 µl of the extracted samples were injected into the LC-MS/MS system. Quantification was performed with MultiQuant 3.0 (Sciex, Darmstadt, Germany) using the internal standard method (isotope-dilution mass spectrometry). Ratios of analyte peak area and internal standard area (y-axis) were plotted against concentration (x-axis), and calibration curves were calculated by least-squares regression with 1/square concentration weighting.

Sample extraction, chromatographic separation, and lipid quantification were performed as described previously^47^.

### DRG primary cultures

The dorsal root ganglia (DRGs) neurons were isolated from mice as previously described^48^. Briefly, twenty to thirty murine dorsal roots ganglia (DRGs) were dissected from thoracal and lumbal spinal segments of one mouse and transferred directly into ice-cold Hanks’ Balanced Salt Solution with CaCl_2_ and MgCl_2_ (Invitrogen, Carlsbad, CA, USA). The DRGs were then incubated with Neurobasal Medium (Invitrogen, Carlsbad, CA, USA) containing 500 U/ml collagenase (Sigma, Deisenhofen, Germany) and 2.5 U/ml dispase II (Roche, Mannheim, Germany) for 75 min at 37 °C. After removal of the collagenase/dispase-solution cells were washed three times with medium containing 10% FCS, 1% penicillin, 1% streptomycin, 1% gentamycin (Invitrogen, Carlsbad, CA, USA) and incubated in 0.05% trypsin for 10 min at 37 °C. After two washing steps, the cells were mechanically dissociated with a 1 ml pipette and plated on poly-L-lysine (Sigma) coated coverslips. After incubation at 37 °C for 2 h, Neurobasal Medium containing glutamine (2 mm), penicillin (100 U/ml), streptomycin (100 μg/ml), B-27 and gentamicin (50 μg/ml) was added to the cells and incubated overnight (modified after^49^). The neurons were used for electrophysiology and Calcium Imaging the next day.

### Calcium Imaging

Calcium-imaging experiments were performed with a Leica calcium-imaging setup, consisting of a Leica DMI 4000 b inverted microscope equipped with a DFC360 FX (CCD) camera, Fura-2 filters, and an N-Plan 10x/0.25 Ph1 objective (all from Leica). Images were taken every 2 s and were processed with the LAS AF-software (Leica). For each experiment, we chose an area with large cell numbers 24 h after preparation (see DRG primary cultures) and monitored 40–110 cells simultaneously. Cells were loaded with 5 μM Fura-2-AM-ester (Biotium), incubated for 45–60 min at 37 °C and washed with ringer solution (145 mM NaCl, 1.25 mM CaCl_2_, 1 mM MgCl_2_, 5 mM KCl, 10 mM D-glucose, and 10 mM HEPES, adjusted to pH 7.3). Baseline measurements were performed with ringer solution at a flow rate of 1–2 ml/min at room temperature. For the activation of TRPV1 we used a solution of 200 nM capsaicin (Sigma), for TRPA1 activation 50 µM allyl isothiocyanat (AITC, Fluka Analytic) and for TRPM8 100 µM L-menthol (Sigma) dissolved in ringer solution. For TRP channel sensitization, cells were incubated for 2 min with different concentrations (50 nM–1000 nM) of (±) 9-HODE (Cayman) or (±) 13-HODE (Cayman) prior to the second stimulus. For control stimulations, the corresponding volume of DMSO (Sigma) was mixed in ringer solution and applied with the same flow rate. 10 µM of the PKA inhibitor H89 (Tocris) or 1 µM of the PKC inhibitor GF109203X (Tocris) was co-incubated with FURA-2 (Biotium) for 45 min. As positive control for PKA activation 40 µM 8-bromo-cAMP (Cayman) and for PKC activation 500 nM bradykinin (Cayman) was used. At the end of each measurement, 50 mM KCl was applied to identify neurons^45^.

### Neuronal calcitonin gene-related peptide release

For the determination of neuronal calcitonin gene-related peptide (CGRP) release, dorsal root ganglia (DRGs) neurons were isolated from mice as previously described^48^. Briefly, DRG cultures were stimulated for 10 min with 1 µM (±) 9-HODE (Cayman) alone or in combination with 400 nM capsaicin (Sigma). Cultures supplemented with capsaicin alone served as control. CGRP concentrations in supernatants were analysed utilizing the rat CGRP Enzyme ImmunoAssay Kit (SPI-Bio, Montigny, France) according to the manufacturer’s instructions. Absorption was measured with the Spectra Max 250 (Molecular Devices, Ismaning, Germany)^50^.

### Electrophysiology

All recordings were carried out at room temperature within 48 hours of plating DRG neurons. Whole-cell voltage clamp recordings were made with an Axopatch 200A amplifier (Molecular Devices) and patch pipettes with resistances of 2–3 MΩ. Pipette solution was 5 mM NaCl, 140 mM KCl, 0.5 mM CaCl_2_, 2 mM MgCl_2_, 5 mM EGTA, 10 mM HEPES, and 3 mM Na_2_ATP, pH 7.2, adjusted with NaOH. The external solution was 140 mM NaCl, 5 mM KCl, 2 mM CaCl_2_, 2 mM MgCl_2_, 10 mM HEPES, and 10 mM D-glucose, pH 7.4, adjusted with NaOH (Sigma). Command protocols were generated and data was digitized with a Digidata 1440A A/D interface with pCLAMP10 software. We used gravity perfusion system connected with perfusion pencil (AutoMate Scientific) to externally apply 9-HODE or capsaicin to the cells^51^.

### Calcium measurement *in vitro*

For studies with heterologously expressed G2A, HEK-293 or COS-1 cells were used. HEK-293 cells were seeded on coverslips and transfected with plasmid containing cDNA for G2A or the empty vector (mock; both obtained from cDNA.org) at a concentration of 1–2 µg DNA/coverslip in DMEM medium containing 10% FCS, 1% penicillin, 1% streptomycin (Invitrogen, Carlsbad, CA, USA) using Turbofect reagent (Thermo Fisher) and following the manufacturer’s instructions. Forty-eight hours later, cells were loaded with FURA-2 (Biotium) for 45 min and measured in calcium imaging experiments as described above.

COS-1 were seeded in white walls-clear bottom 96-well plates and transfected with a plasmid encoding a calcium-sensitive bioluminescent fusion protein between aequorin and GFP^52^ together with plasmid containing cDNA for G2A or control DNA (empty vector, mock) at a concentration of 50 ng/well by using FuGENE 6 reagent (Promega) following manufacturer’s instructions. Forty-eight hours later, cells were loaded with 5 µM coelenterazine *h* (Invitrogen) in HBSS containing 1.8 mM CaCl_2_ and 10 mM glucose for 2 h at 37 °C. Measurements were performed by using a luminometric plate reader (Flexstation 3). The area under each calcium transient was calculated by using SoftMaxPro software and expressed as area under the curve (AUC)^53^.

### Quantitative Real-Time PCR

Lumbal DRGs were dissected from mice at indicated time points, and RNA was extracted using the mirVana miRNA Isolation Kit (Ambion, Life Technologies). Reverse transcription and real-time PCR were performed using the TaqMan system^54^ (Life Technologies) and evaluated with the ΔΔC(T) method as described previously^55^. For G2A and TRPV1, assay primers for the TaqMan system (ThermoFisher) were used.

### Data Analysis and Statistics

All data are presented as mean ± SEM. To determine statistically significant differences in all behavioral experiments, Two-way ANOVA for repeated measures was used, followed by Bonferroni’s *post hoc* correction using GraphPad Prism 6. For *in vitro* experiments comparing only two groups, Student’s *t* test was carried out and for comparing more than two groups, One-way ANOVA followed by Bonferroni’s *post hoc* correction was used. A *p* value of <0.05 was considered statistically significant.
